# Impact of solid surface hydrophobicity and micrococcal nuclease production on *Staphylococcus aureus* Newman biofilms

**DOI:** 10.1038/s41598-020-69084-x

**Published:** 2020-07-21

**Authors:** Abigail M. Forson, Henny C. van der Mei, Jelmer Sjollema

**Affiliations:** University of Groningen, University Medical Centre Groningen, Department of Biomedical Engineering, A. Deusinglaan 1, 9713 AV Groningen, The Netherlands

**Keywords:** Bacteria, Biofilms, Microbial communities

## Abstract

*Staphylococcus aureus* is commonly associated with biofilm-related infections and contributes to the large financial loss that accompany nosocomial infections. The micrococcal nuclease Nuc1 enzyme limits biofilm formation via cleavage of eDNA, a structural component of the biofilm matrix. Solid surface hydrophobicity influences bacterial adhesion forces and may as well influence eDNA production. Therefore, it is hypothesized that the impact of Nuc1 activity is dependent on surface characteristics of solid surfaces. For this reason, this study investigated the influence of solid surface hydrophobicity on *S. aureus* Newman biofilms where Nuc1 is constitutively produced. To this end, biofilms of both a wild-type and a nuc1 knockout mutant strain, grown on glass, salinized glass and Pluronic F-127-coated silanized glass were analysed. Results indicated that biofilms can grow in the presence of Nuc1 activity. Also, Nuc1 and solid surface hydrophobicity significantly affected the biofilm 3D-architecture. In particular, biofilm densities of the wild-type strain on hydrophilic surfaces appeared higher than of the mutant nuc1 knockout strain. Since virulence is related to bacterial cell densities, this suggests that the virulence of *S. aureus* Newman biofilms is increased by its nuclease production in particular on a hydrophilic surface.

In general, bacteria prefer living in communities at a surface rather than as single individuals in their aqueous surroundings. It is therefore not surprising that the biofilm mode of growth is paramount to the survival of microorganism in both industrial and medical settings. Biofilms are notorious for causing food-borne diseases due to their formation on food and food factory equipments^1^ but on the other hand can be beneficial in waste water treatments^2^. Biofilms and in particular biofilms of* Staphylococcus aureus* that establish on medical implants still remain the underlying factor for infection recurrence and refractory response to conventional antibiotic treatments^3–5^.

A biofilm is described as a bacterial community wrapped in a self-produced matrix of extracellular polymeric substances (EPS). EPS generally consist of polysaccharide intercellular adhesin (PIA) or poly-N-acetylglucosamine (PNAG), proteins, RNA, lipids and extracellular DNA (eDNA), depending on the bacterial strain^6,7^. eDNA on the surface of planktonic *S. aureus* has been shown to improve adhesion as well as stabilize *S. aureus* biofilm structure at low pH^8^. During the early stages of *S. aureus* biofilm formation, a short period of heightened micrococcal nuclease production occurs which has been described to result in a first round of bacterial dispersal^9^. As the biofilm matures, bacteria continue to grow and produce EPS. This is accompanied by a second round of cell detachment through the peripheral and in-depth expression of the accessory gene regulator (*agr*) quorum sensing system once a critical mass is reached^10,11^.

*Staphylococcus aureus* independently expresses two forms of micrococcal nucleases, the excreted Nuc1 and the membrane bound Nuc2. Nuc1 expression is regulated by the SaeRS two-component system^12–14^. However, no regulation mechanism has been identified for Nuc2 expression^15^. Nuc1 is described as the principal enzyme responsible for *S. aureus* nuclease activity in vitro^16,17^ and is therefore the focus of this study. This enzyme utilizes Ca^2+^ for its endo- and exo-5′ phosphodiesterase activity against both DNA and RNA to give 3-mono- and di-nucleotides, making biofilm eDNA a suitable target^18^. An induced expression of Nuc1 in *S. aureus* biofilms is described to result in decreasing biofilm biomass due to its ability to cleave eDNA^19,20^.

Bacteria have been shown to adhere and behave differently on hydrophobic and hydrophilic surfaces^21^ making solid surface hydrophobicity pivotal for biofilm formation. In earlier studies, bacteria that managed to attach to a Pluronic F-127 coating on silicone rubber adhered with a lower adhesion force in comparison to uncoated silicone rubber. This low adhesion force conferred a semi-planktonic state on the bacteria which was characterized by a lack of EPS production under biofilm-forming conditions^22^. Moreover, production of PNAG and eDNA by *S. aureus* decreased with increasing adhesion force on different biomaterials^23^.

Since eDNA is a vital structural component of EPS which in turn can be influenced by solid surface hydrophobicity, we hypothesize that solid surface hydrophobicity affects the impact of Nuc1 activity in biofilms. Identifying a possible connection between solid surface hydrophobicity and the vulnerability of biofilms to Nuc1 activity may provide useful clues in designing biomaterials. Therefore, this study aimed at investigating the impact of varying solid surface hydrophobicities on biofilm formation of *S. aureus* Newman WT and its isogenic mutant ∆*nuc1*. *S. aureus* Newman which constitutively produces Nuc1 as result of a point mutation in the SaeRS two component system^24^. In addition, a combination of biofilm analysis methods were utilized to elucidate the combined effect of variable solid surface hydrophobicities and Nuc1 production on biofilm structure and properties in vitro. Glass (hydrophilic), silanized glass (hydrophobic) and a polyethylene oxide (PEO)-brush-like coating (Pluronic F-127)^25^ on silanized glass (hydrophilic) were utilised as solid surfaces.

## Results

### Effect of solid surface hydrophobicity on bacterial adhesion

**Figure 1:**
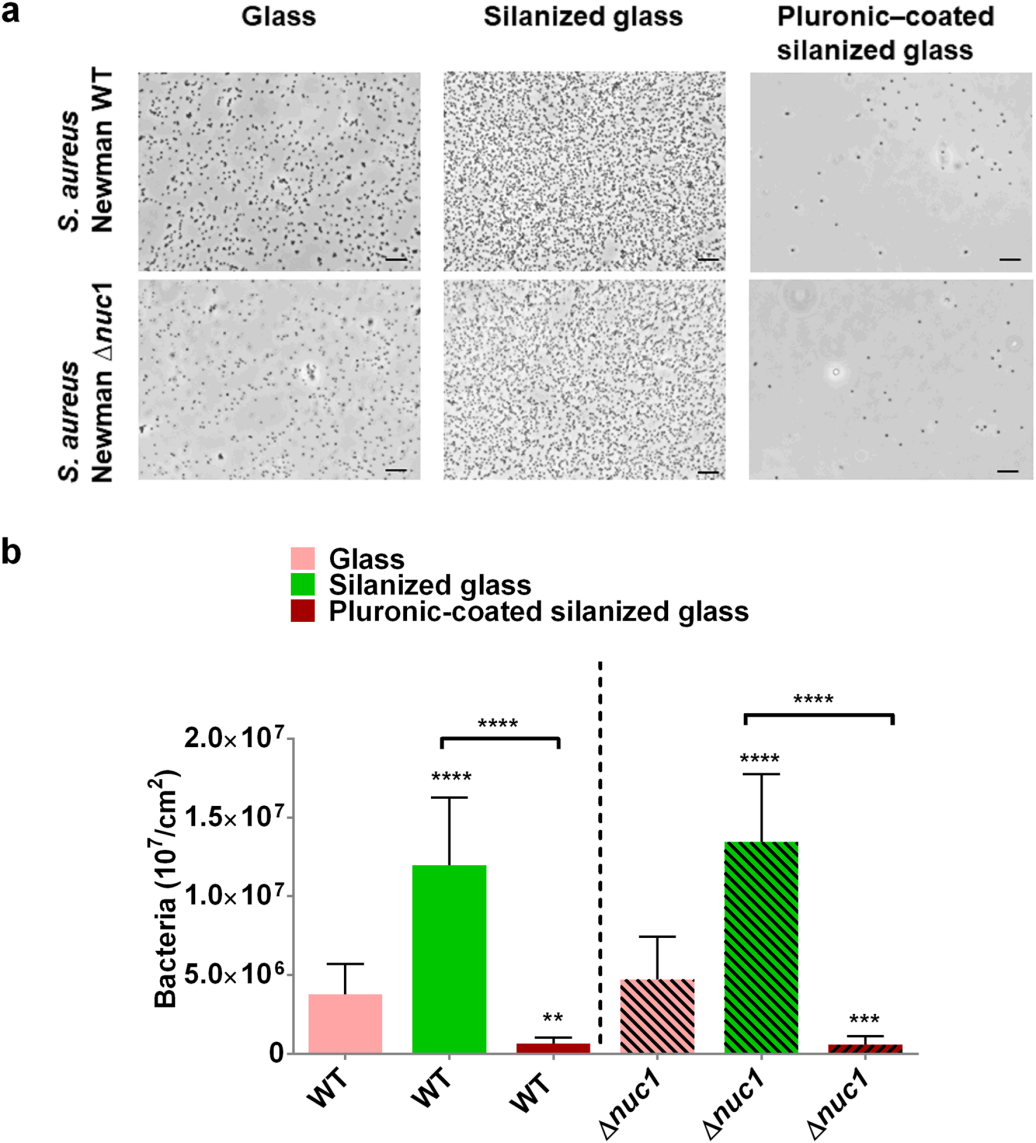
The impact of solid surface hydrophobicity on bacterial adhesion. (**a**) Phase contrast micrographs. Scale bar = 10 µm. (**b**) Number of adhered *S. aureus* Newman WT and *S. aureus* Newman ∆*nuc1* per unit surface area on glass, silanized glass and Pluronic F-127-coated silanized glass. Bars indicate the average number of adhered bacteria after 1 h adhesion under rotation at 150 RPM with three different bacterial cultures. Error bars show the standard deviation. Statistical significance between bacterial numbers on different surfaces by identical strains are indicated with asterisks, **P ≤ 0.01; ***P ≤ 0.001; ****P ≤ 0.0001.

Adhesion of *S. aureus* Newman and its isogenic *nuc1* mutant were first investigated on glass (water contact angle 16° ± 21°), silanized glass (water contact angle 96° ± 8°) and Pluronic F-127-coated silanized glass (water contact angle ≤ 25 ± 1)^26^. Planktonic cultures of both staphylococcal strains were allowed to adhere to the solid surfaces for 1 h after which adhered bacteria were imaged with a phase contrast microscope. The obtained results revealed a lack of significant difference between adhesion of the WT and mutant strain on the same solid surface (Fig. 1a, b). Both bacterial strains showed the highest affinity for silanized glass and the lowest number of adhered bacteria was found on Pluronic F-127-coated silanized glass.

### Nuclease activity in planktonic cultures and biofilms

**Figure 2:**
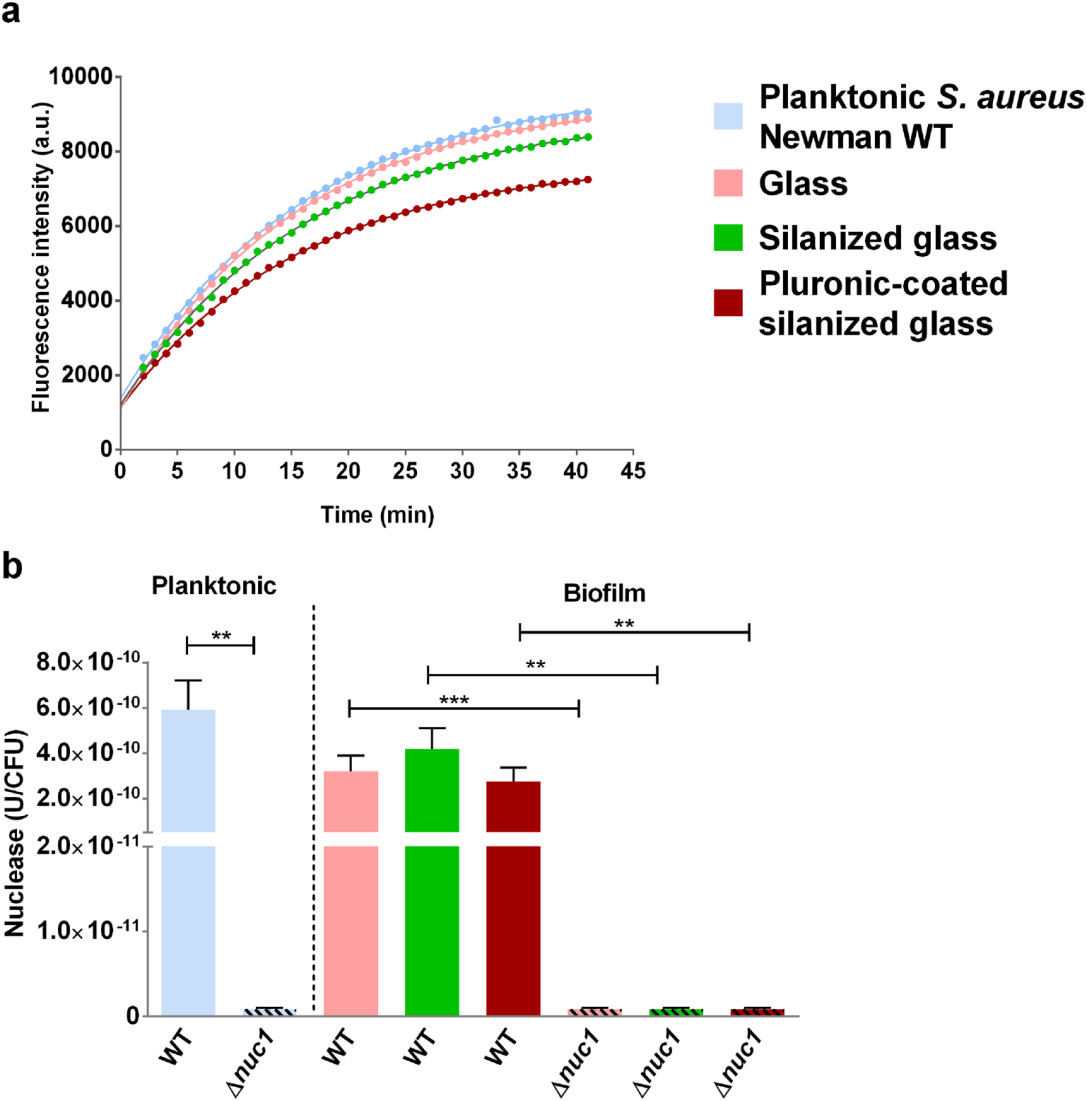
Nuclease activity is reduced during biofilm formation. (**a**) Fluorescence intensity of a FRET-based DNA probe assay in 20 h old planktonic cultures and biofilms of S. aureus Newman WT on glass, silanized glass and Pluronic F-127-coated silanized glass. Dots indicate the mean of 4 different planktonic cultures and 6 biofilms grown with three different bacterial cultures. Lines are least square fits of Eq. (1) to the measured intensity data. Error bars are eliminated for easy readability. (**b**) Nuclease concentration per CFU was determined in 20 h old planktonic cultures and biofilms of S. aureus Newman WT and *S. aureus* Newman ∆nuc1 grown on glass, silanized glass and Pluronic F-127-coated silanized glass. Bars indicate the mean of 4 different planktonic cultures and 6 biofilms grown with three different bacterial cultures. Error bars show the standard error of the mean. Statistical significance between WT and mutant strain on the same solid surfaces is indicated with asterisks, **P ≤ 0.01; ***P ≤ 0.001.

Nuclease activity in planktonic cultures of both staphylococcal strains as well as in the biofilms grown on glass, silanized glass and Pluronic F-127-coated silanized glass were measured with a Förster resonance energy transfer (FRET)-based DNAse assay (Fig. 2a). The data obtained revealed that Nuc1 activity persisted during biofilm formation but was decreased by twofold, 1.5-fold and 2.2 fold per CFU respectively on glass, silanized glass and Pluronic F-127-coated silanized glass in comparison to planktonic WT (Fig. 2b). Note that the nuclease activity for the mutant strain was almost zero.

### Effect of solid surface hydrophobicity and Nuc1 production on biofilm thickness and structure

**Figure 3:**
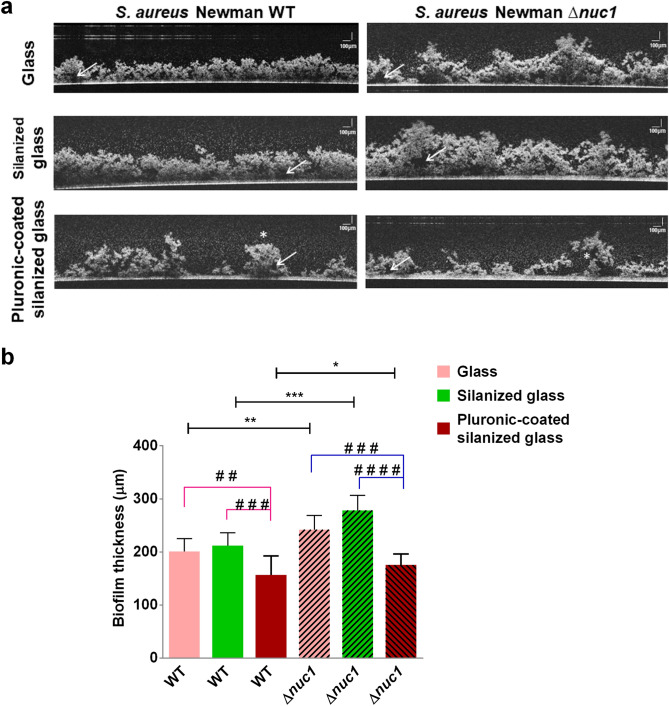
Synergistic effect of nuclease activity and different solid surface hydrophobicities on biofilm structure and thickness. (**a**) 2D OCT images of 20 h biofilms grown on glass, silanized glass and Pluronic F-127-coated silanized glass. Arrows indicate voids in the biofilms and stars indicate island-like biofilms. (**b**) Biofilm thickness determined with OCT on surfaces with different hydrophobicities. Bars represent the mean thickness of 8 biofilms grown with 4 different bacterial cultures. Statistical significance between biofilms formed by WT and mutant strain on identical surfaces are indicated with asterisks, *P ≤ 0.05; **P ≤ 0.01; ***P ≤ 0.001. Statistical significance between biofilms formed on different substrates by identical strains are indicated with hashtags, ^# #^*P* ≤ 0.01, ^# # #^*P* ≤ 0.001, ^# # # #^*P* ≤ 0.0001.

Optical coherence tomography (OCT) was employed to acquire cross sectional images of *S. aureus* Newman WT and *S. aureus* Newman ∆*nuc1* biofilms on all tested solid surfaces after 20 h of growth. As can be seen in Fig. 3a, biofilms appeared as white reflective spots while fluid-filled pores or channels, also referred to as voids, appeared as dark transparent spots on the OCT images (shown by arrows in Fig. 3a). The 2D OCT images showed that the surface of the biofilm from the mutant strain was rougher than that of the WT strain on glass and silanized glass. The outer surface roughness of the biofilm was similar for both staphylococcal strains grown on the Pluronic F-127-coated silanized glass and showed more island like structures (indicated by stars in Fig. 3a) than on the other two surfaces. The biofilm thickness, as determined with a 2D analysis software, revealed a significantly thinner biofilm of the WT strain than the mutant strain on all tested solid surfaces (Fig. 3b and Fig. S3). The biofilm on Pluronic F-127-coated silanized glass was significantly thinner with respect to silanized glass and glass (Fig. 3b and Fig. S3). However, there was no significant difference between the thickness of biofilms formed on glass and silanized glass (Fig. 3b).

### Effect of Nuc1 production and solid surface hydrophobicities on biofilm density, eDNA and EPS polysaccharide

**Figure 4:**
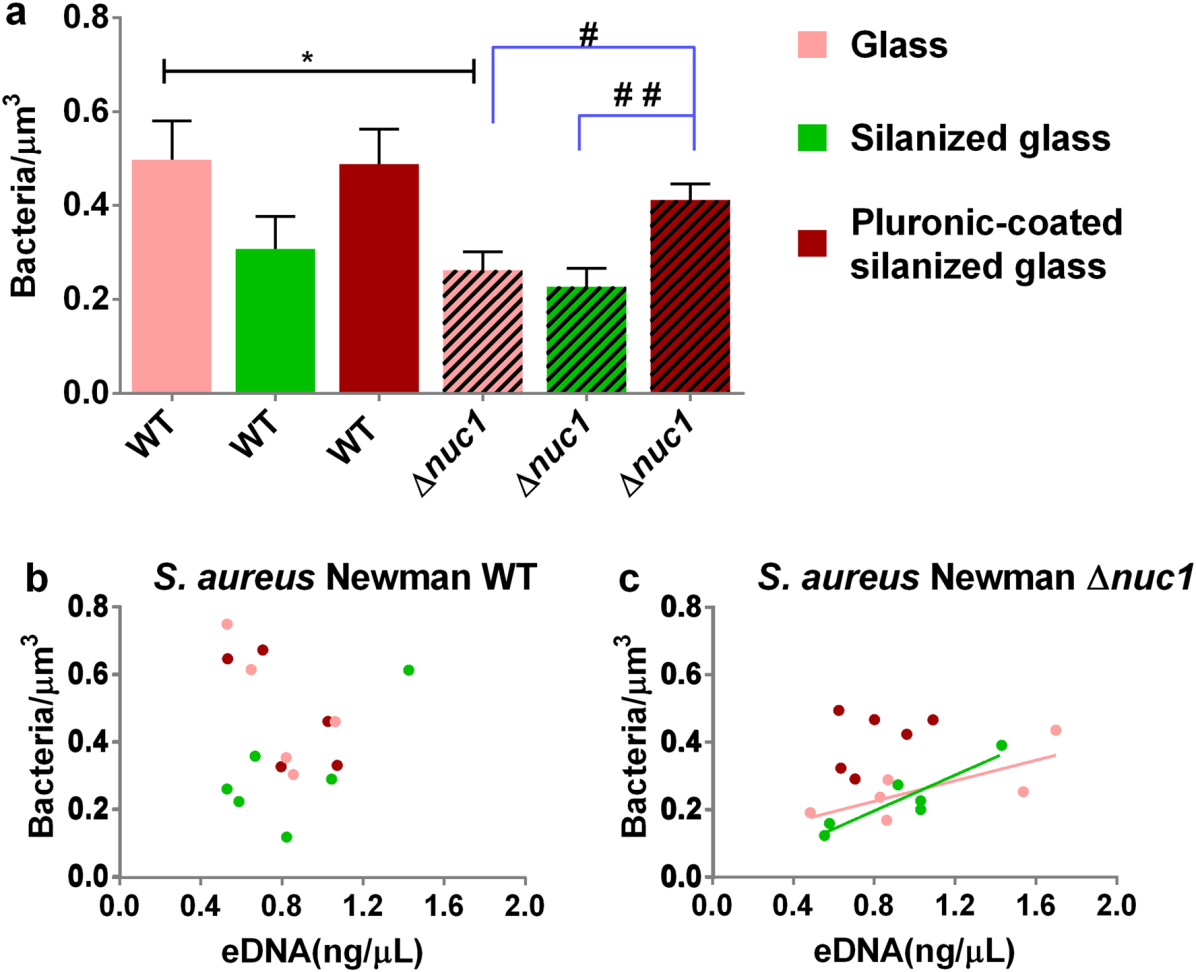
Evaluation of substrate surface chemistry and Nuc1 production on biofilm properties. (**a**) Bacterial density of 20 h biofilms grown by *S. aureus* Newman WT and S. aureus Newman ∆nuc1 on glass, silanized glass and Pluronic F-127-coated silanized glass Bars represent the mean of six biofilms grown with 3 different bacterial cultures. Error bars are the standard error of the mean. Statistical significance between biofilms formed by WT and mutant strain on identical surfaces are indicated with asterisks, *P ≤ 0.05. Statistical significance between biofilms formed on different substrates by identical strains are indicated with hashtags, ^#^*P* ≤ 0.05; ^##^*P* ≤ 0.01. (**b**), (**c**) Bacterial density as a function of eDNA concentration in biofilm. Lines indicate a significant correlation between uncleaved eDNA content and biofilm density on glass and silanized glass based on a Pearson correlation test. Dots represent eDNA concentration/density data pairs from the same sample.

The results revealed that the bacterial density of the WT biofilm is higher than that of the mutant strain on glass (Fig. 4a). The bacterial density of mutant biofilms grown on the Pluronic-coated silanized glass was significantly higher than their counterparts on glass and silanized glass (Fig. 4a). PicoGreen staining showed equivalent eDNA content in biofilms formed by both strains on all tested surfaces (see Supplementary Fig. S2a). A Pearson correlation test showed no significant relation between the eDNA concentration and density in biofilms on glass (r = − 0.72, *P* = 0.16), silanized glass (r = 0.72, *P* = 0.11) and Pluronic F-127-coated silanized glass (r = − 0.72, *P* = 0.17, Fig. 4b) for the WT strain. A Pearson correlation test revealed a positive relation between the concentration of eDNA and bacterial density of biofilms formed on glass (r = 0.75, *P* = 0.08) and silanized glass (r = 0.91, *P* ≤ 0.01, Fig. 4c) only for the mutant strain. There were no significant differences between the CFU and EPS production of both staphylococcal strains on all tested solid surfaces (Fig. S2, b and c).

## Discussion

As shown in Fig. 1 solid surface hydrophobicity impacted bacterial adhesion. Irreversible adhesion is suspected to have occurred via the hydrophobic effect due to the removal of interfacial water between the bacterium and the solid surface and hydrogen bonding on the hydrophobic and hydrophilic surfaces respectively^27^. The Pluronic F-127 coating was effective in restricting adhesion because an approaching bacterium will press the stretched hydrophilic PEO chains towards the hydrophobic PPO chains and subsequently be repelled due to steric hindrance^28^. This has been found to be valid for other strains as well^21,26,29,30^. Additionally, the presence of a higher number of adhered bacteria on silanized glass (Fig. 1) may imply a higher number of hydrophobic than hydrophilic patches on the surface of the *S. aureus* Newman strains, fostering an energetically more favorable condition on hydrophobic surfaces.

It is known that DNAse may affect adhesion as was shown by a significant reduction of *S. aureus* and *Pseudomonas aeruginosa*, preventing biofilm formation for 14 h^31^. Nucleases in our assays did not show these effects since no differences in numbers of adhered bacteria were found between the wild type and nuclease deficient mutants (Fig. 1), indicating that cell surface associated eDNA was not affected by the expression of nuclease. In other studies, glucose supplementation to boost biofilm formation resulted in a tenfold decreased production and activity of Nuc1^19,32^. In our biofilm assay, it was observed that Nuc1 production was reduced in biofilms with respect to their planktonic counterparts even without glucose supplementation (Fig. 2).

*S. aureus* Newman has been classified as a poor biofilm former due to a point mutation and consequent constitutive activation of the SaeRS two-component system effecting a constant *nuc1* expression^24,33^. As expected based on literature^24^ we did not find differences in nuclease production per CFU at the respective substrates as a result of the constitutive expression of *nuc1*. In this study it was found however that biofilm formation by the *S. aureus Newman* strain was not hindered by the constitutive Nuc1 production (Figs. 2, 3). The minimum biofilm thickness recorded in this study (160 ± 37 μm) was higher than the 30 ± 2 μm thickness recorded for *S. aureus* MFP03^34^. In this study, the formation of thicker biofilms by the *S. aureus* Newman strain compared to literature may be partly due to stronger adhesion under the dynamic conditions employed in our biofilm assay^35^. In an earlier report by Kiedrowski et al.^19^, the biomass of biofilms formed by *S. aureus* Newman was comparable to that of *S. aureus* USA300 and *S. aureus* TCH151 and higher than that of *S. aureus* COL. These results may also suggest that the eDNA in the biofilm is stabilized, possibly via interactions with DNA-binding proteins like Eap^36^ and in particular SaeP^36^, which is expressed as an auxiliary protein in the SaeRS two component system^37^.

We further focused on the synergistic effect of solid surface hydrophobicity and Nuc1 activity on biofilms of *S. aureus* Newman and its isogenic ∆*nuc1* mutant. One major finding in this study is that solid surface hydrophobicity affects the internal configuration of biofilms as evidenced by the changes in density with and without Nuc1 activity (see Fig. 4). eDNA is essential for biofilm structure^38^, acting as an electronegative string that tether bacteria surrounded by net positively charged matrix proteins and PIA^8^. In essence, eDNA in *S. aureus* Newman WT biofilms will be cleaved due to Nuc1 activity while eDNA in the mutant strain biofilms remain intact (Fig. 2). This was shown earlier in agarose gels where eDNA from wild type MRSA bacteria appeared as a smear of mainly low molecular weight DNA fragments, whereas the eDNA from mutant strains were of high molecular weight similar to purified genomic DNA^19^. Employing a ball and stick model to represent the 3D net-like architecture of a biofilm^8^, eDNA can be represented by sticks. Likewise, bacteria connected to positively charged proteins and PIA are represented by balls. Nuc1 activity will cleave eDNA which will result in relative short “sticks” in the ball-and-stick model. Lack of Nuc1 activity in the mutant strain biofilms will result in maintaining the connecting eDNA (sticks) which will in turn lead to an expansion of the biofilm net-like architecture as related to the WT strain. First, this expanded state is evidenced by the rougher outer surface of the biofilms formed by the mutant strain on glass and silanized glass (Fig. 3a). Second, the expanded state of the biofilm resulted in an increase in biofilm thickness (Fig. 3b) as well as a decrease in density of the mutant biofilms which was significant on glass but not on silanized and Pluronic F-127-coated solid surfaces (Fig. 4a). Also, for the WT strain the density was not affected by higher eDNA content as was the case for the mutant strain on glass and silanized glass, evidenced from significant positive correlations between eDNA content and bacterial cell density on glass and silanized glass (Fig. 4b, c). This correlation was not observed for Pluronic F-127 coated surfaces, signifying again that surface characteristics affected the net-like architecture of biofilms. These differences are also partly in line with increasing amounts of EPS with decreasing hydrophobicity, specifically eDNA and PNAG which are necessary for cell–cell interactions^23^. Thus, nucleases in particular modulate the EPS binding capacity in biofilms.

Bacterial biofilm density has earlier been reported to range between 0.2 and 0.4/μm^3^ which is relatively low with respect to a closed packing that would lead to a bacterial density of 1.4/μm^3^ ^39^. This indicates that *S. aureus* Newman biofilms, the bacterial density of which range up to 0.5/μm^3^, are relatively dense, specifically on hydrophilic surfaces like glass and Pluronic-coated salinized glass. Since bacterial density has earlier been linked to the expression of the *agr* system which regulates the expression of several *S. aureus* virulence genes^9,11^, it is suggested that Nuc1 production in combination with the surface hydrophobicity influences the virulence associated with *S. aureus* biofilms on implants. Mukherjee et al.^40^ also predicted that an increase in density may increase the production of an autoinducer of the *agr* system. In addition to this, the expression of RNA III, the main effector of the *agr* system, was seen to be elevated with increasing cell density in vegetations after *S. aureus* infection in an experimental endocarditis study^41^. In *S. aureus* biofilms, *agr* quorum sensing improves α-toxin production which is harmful to immune cells and promotes cell detachment independent of Nuc1 activity^9,19,42,43^. Although results from this in vitro study does not depict a perfect representation of events in vivo, the increase in biofilm density due to Nuc1 activity on hydrophilic surfaces, taken together with earlier findings of increased *agr* expression with increasing bacterial density, strongly suggests a higher virulence of the *S. aureus* strains on hydrophilic solid surfaces.

In summary, this study identified biofilm formation occurring in the presence of Nuc1 activity (Figs. 2, 3). Lack of Nuc1 activity resulted in an expansion of the biofilm net-like architecture due to the presence of uncleaved eDNA. Nuc1 activity caused significant density differences between the WT and mutant strain in particular on the hydrophilic glass (Fig. 4). Augmented *agr* expression as expected from increasing density suggests that the virulence of biofilm associated infections caused by *S. aureus* Newman is affected by the production of nuclease in particular on a hydrophilic surface like glass.

## Materials and methods

### Solid surfaces modifications

Round cover glasses (15 mm diameter, Marienfeld, Lauda-Königshofen, Germany) were cleaned by sonicating for 5 min in 2% RBS in a bath sonicator (Salm en Kipp b.v., Breukelen, The Netherlands). This was followed by washing 3 times in demineralized water and a subsequent 5 min incubation in methanol (CH_3_OH, EMPLURA, Darmstadt, Germany). The cover glass was then washed 3 times in demineralized water and kept submerged until used for glass silanization (i).

### Glass silanization

To activate glass for the silanization step, clean glass cover slips were incubated in a 1:1 mixture of hydrochloric acid (HCl, 12 M, EMSURE, Darmstadt, Germany) and methanol for 40 min and rinsed at least 5 times with ultrapure water (18.2 MΩ). The cover glass was subsequently incubated for 40 min in sulfuric acid (H_2_SO_4_, 95–97%, EMSURE, Darmstadt, Germany) then washed 5 times with ultrapure water. After this, the glass was placed in gently boiling ultrapure water for 1 h and allowed to cool at room temperature. The cover glass was dried with filter sterilized air and carefully placed in a dry petri dish. A small container with 50 µL of propyltrichlorosilane (CH_3_CH_2_CH_2_SiCl_3_, Sigma, Saint Louis, USA,) was placed next to the activated cover glass and kept under vacuum overnight.

### Pluronic F-127 coating

Silanized glass was coated with Pluronic F-127 using a modified protocol based on the studies of Nejadnik et al.^25^. In summary, silanized glass was sterilized in 70% ethanol and rinsed in sterile demineralized water. The glass was then placed in a 24-wells plate (Greiner bio-one, USA) and incubated in filter sterilized Pluronic F-127 (MW 1,200 g/mol, PEO_100_-PPO_70_-PEO_100,_ 0.05%, Sigma, Saint Louis, USA) solution in phosphate buffered saline (PBS, 10 mM potassium phosphate, 150 mM NaCl, pH 7.0) for 1 h at room temperature. Excess Pluronic F-127 was removed by carefully washing 1 time with PBS.

### Water contact angle measurement

Water contact angles of glass and silanized glass were measured with the sessile drop technique using a home-made contour monitor. Ultrapure water droplets of 2 μL were placed on surfaces and the contact angle was measured with an image analysis software program (MATLAB, MathWorks, Natick, MA, USA). To accurately predict hydrophobicity on the large surface area of glass and silanized glass, contact angles were calculated as the mean of measurements from 9 different positions on silanized glass and glass.

### Bacterial strains and culture

*S. aureus* Newman and its isogenic *S. aureus* Δ*nuc1* were kindly donated by Prof. McNamara (Department of Internal Medicine, University of Illinois, USA) and used for all the experiments in this study. *S. aureus* Newman is an MSSA strain which was isolated from a case of tubucular osteomyelitis in human^44^. The *S. aureus* Newman ∆*nuc1* was constructed by Kiedrowski et al.^19^ using the Targetron Gene Knockout system. Single colonies of the strains were obtained by aerobic culturing on blood agar plates for 24 h at 37 °C. To make a preculture, one colony of each strain was inoculated in 10 mL of Tryptone Soya Broth (TSB, OXOID, Basingstoke, UK) and cultured for 24 h at 37 °C. The preculture was transferred to 200 mL TSB and grown for 17 h at 37 °C for the main culture.

### Multi-well plate biofilm assay

The bacterial cells were harvested from the main culture by centrifugation (6,250 g 5 min, 10 °C) and washed twice in PBS. Bacteria were sonicated 3 × 10 s (Vibra Cell Model 375, Sonics and Materials Inc., Danbury, CT, USA) in an ice-water bath to disrupt bacterial aggregates and enumerated in a Bürker-Türk counting chamber. The bacterial suspension was diluted to a concentration of 1 × 10^9^ mL^−1^ with sterile PBS and transferred to a 24-wells plate containing the solid surfaces (glass, silanized glass, and Pluronic F-127 coated silanized glass). The bacteria were allowed to adhere for 1 h under rotation in a shaking incubator at 150 RPM at 37 °C after which unattached bacteria were removed by washing with sterile PBS. To evaluate the amount of *S. aureus* Newman WT and Newman ∆*nuc1* that adhered on all tested surfaces, a phase contrast microscope (Olympus) was utilized to image the number of adhering bacteria per unit area. The bacteria were counted manually or with Fiji^45^. To grow biofilms, TSB was added to each well containing a particular solid surface with adhering bacteria and incubated for 20 h (37 °C, 80 RPM). After the incubation period, growth media was removed without exposing the biofilms to air and washed twice with sterile PBS. This was done before further analysis of the biofilms unless otherwise stated.

### Nuclease activity assay

Nuclease activity was measured in 20 h old planktonic cultures or biofilms of *S. aureus* Newman WT and *S. aureus* Newman ∆*nuc1*. The biofilms were resuspended in their growth medium to aid additional release of Nuc1 from the biofilm EPS. All fluid samples were diluted 1,000 times in 10 mM Tris–HCl (pH 8, 10 mM CaCl_2_). 25 µL of each diluted suspension was added to 150 µL of Tris–HCl supplemented with calcium in a black 96 well-plate with clear bottom (CELLSTAR, 655,087, Greiner bio-one, USA). 25 µL of a 2 µM working stock of a FRET-based DNA probe (5′-6FAM TTTTTTTTTTBHQ1, Sigma, Saint Louis, USA) was added and fluorescence intensity was immediately measured at 1 min intervals during 39 min with a Fluorstar Optima (BMG LABTECH, Offenburg, Germany) plate reader at excitation 485 nm and emission at 520 nm. Wells containing biofilm suspension and Tris–HCl buffer but without the DNA probe were used as a blank as well as a negative control. As a positive control, biofilm suspension was replaced with 25 µL of 0.001 U/mL purified staphylococcal nuclease (Sigma, Saint Louis, USA) reaching a final concentration of 0.000125 U/mL. A calibration curve was performed using purified staphylococcal nuclease at concentrations 0, 0.0001, 0.0002, 0.0004, 0.0008 and 0.001 U/mL (Fig. S1a). Data obtained for *S. aureus* Newman WT biofilms and purified staphylococcal nuclease were fitted to Eq. (1):

1 $$ y\left( t \right) = c + a*\left( {1 - e^{ - bt} } \right) $$

where y(t) is the fluorescence intensity, *c*, *a* and *b* are fitting parameters (c = 2,000, a = 1,000 and b = 10) and t is time in minutes. The initial rate at which Nuc1 cleaves the DNA probe was determined from the first derivative at t = 0, being a * b from Eq. (1). The initial rate of activity of the data obtained from Nuc1 mutant strain were determined using a linear regression of the first five measurements due to very low signal. All data fittings were executed in GraphPad Prism version 6 (GraphPad Software, La Jolla California, USA).

### Biofilm analysis with optical coherence tomography (OCT)

*S. aureus Newman* WT and *S. aureus Newman* ∆*nuc1* biofilms were imaged with an OCT Ganymede II (Thorlabs Ganymade, Newton, New Jersey, USA) device using a white light beam of 930 nm. Ten 2D images were taken using the refractive index of water, 1.33, a field of view of 4 mm and 2 µm pixels in the vertical direction. The average thickness of each 2D image was determined with a 2D OCT analysis software (Thorlabs, Newton, New Jersey, USA). In brief, a grey value threshold was determined that separates the biofilm from the surrounding liquid based on the grey value distribution of the particular image. The upper contour line of the biofilm was defined as those pixels that were connected to the bottom of the biofilm by pixels with grey values higher than the grey-value threshold. The software determined the bottom contour line of the biofilm by connecting six points which were manually placed at the solid surface-biofilm interface. The biofilm thickness could then be determined based on the average number of pixels between the bottom and upper contour line^39^.

### Colony forming units

Biofilms formed by *S. aureus* Newman and its isogenic Nuc1 mutant were washed twice and resuspended in 1 mL sterile PBS. The biofilm suspension was sonicated for 5 min in a bath sonicator (Salm en Kipp b.v., Breukelen, The Netherlands). After which vigorous pipetting was used to further suspend the biofilm. A serial dilution of the biofilm suspension was made in sterile PBS and 100 μL plated on TSB agar plates followed by incubation at 37 °C overnight.

### Bacterial density

The bacterial suspensions were diluted 100 times and counted in a Bürker-Türk counting chamber. The values obtained for the total number of bacteria per biofilm were used in calculating the total bacterial density of the biofilms per unit volume by dividing the total cell count by the biofilm volume, calculated through multiplying the biofilm area by its thickness as defined in Eq. (2):

2 $$ Total\, bacterial\,density \left( {\mu m^{ - 3} } \right) = \frac{Total \,bacterial \,count }{{average\, biofilm\, thickness \left( {\mu m} \right)*area\, of\, biofilm \left( {\mu m^{2} } \right)}} $$

### PicoGreen staining

eDNA was quantified as described by Tang et al.^46^. Briefly, growth media of 20 h old *S. aureus* Newman WT and *S. aureus* Newman ∆*nuc*1 were removed and the biofilm was washed with sterile PBS. The biofilms were suspended by gently pipetting up and down. 100 µL of the biofilm suspension was mixed with 100 µL freshly prepared PicoGreen solution (1 µL PicoGreen dye in 199 µL TE buffer (10 mM Tris–HCl, 1 mM EDTA, pH 8) in a black 96-well plate with a clear bottom and incubated for 4 min at room temperature before measuring the fluorescent intensity with a Fluorstar Optima plate reader (excitation/emission 485 nm/520 nm). A calibration curve was performed using a concentration range of 0–1,000 ng/mL of λ DNA (Fig. S1b).

### Calcofluor white staining for EPS polysaccharide

250 µL of a 50 mM stock solution of fluorescent brightener 28 (Calcofluor white, Sigma Aldrich, C_40_H_44_N_12_O_10_S_2_ Sigma, Saint Louis, USA) that had been diluted 250X in PBS was added to wells containing biofilms of *S. aureus* Newman WT and *S. aureus* Newman ∆*nuc1*. The biofilms were incubated in darkness for 30 min at room temperature. After this the Calcofluor white solution was carefully removed and the biofilm was washed with sterile PBS. The biofilms were gently mixed by pipetting and transferred to a black 96-well plate with clear bottoms. Fluorescent intensity was measured with a Fluorstar fluorescent plate reader with excitation and emission wavelengths at 355 nm and 490 nm respectively.

### Statistical analysis

All biofilms were grown in duplicate and repeated with three or four different cultures. Statistical differences between experimental groups on identical and dissimilar solid surfaces were analysed with a Students t-test and ANOVA test respectively, using GraphPad Prism version 6 (GraphPad software, La Jolla California, USA). Differences were considered significant if *P* ≤ 0.05.

## Data availability

The datasets generated during and/or analysed during the current study are available from the corresponding author on reasonable request.

## Supplementary information

Supplementary Information.
