# Supplementary material for: Impact of solid surface hydrophobicity and micrococcal nuclease production on Staphylococcus aureus Newman biofilms
Source: Sci Rep. 2020 Jul 21;10:12093. doi: 10.1038/s41598-020-69084-x (PMC7374737; doi:10.1038/s41598-020-69084-x)
Supplement: Supplementary file 1 — Supplementary Information. [file 41598_2020_69084_MOESM1_ESM.docx]

**Supplementary Data**

**Impact of Solid Surface Hydrophobicity and Micrococcal Nuclease Production on *Staphylococcus aureus* Biofilms**

Abigail M. Forson, Henny C. van der Mei, Jelmer Sjollema*

*University of Groningen, University Medical Centre Groningen, Department of Biomedical Engineering-FB40, A. Deusinglaan 1, 9713 AV, Groningen, Netherlands*

*Correspondence should be addressed to Jelmer Sjollema; [j.sjollema@umcg.nl](mailto:j.sjollema@umcg.nl)

Figure S1. Calibration curve for nuclease activity (a) and calibration curve for PicoGreen staining (b). A FRET- based DNase Assay was used to infer the initial cleaving rate of commercially available staphylococcal nuclease of known concentrations (a). PicoGreen stain was used to determine the fluorescence intensity of known concentrations of λ DNA after 4 min incubation in PicoGreen solution (b). Data points represent the average of 2 independent measurements. Error bars indicate the SEM.

Figure S2. Evaluation of substrate surface chemistry and Nuc1 production on biofilm properties. (a) eDNA concentration determined by PicoGreen staining, (b) EPS polysaccharides measured by Calcofluor white staining, (c) CFU of 20 h biofilms of S. aureus Newman WT and S. aureus Newman ∆nuc1 in TSB on glass, silanized glass and Pluronic F-127-coated silanized glass. Bars represent the mean of six biofilms s from 3 different bacterial cultures. Error bars are the standard error of the mean.

Figure S3. Biofilm thickness determined with OCT on surfaces with different hydrophobicities. Circles/squares represent the mean thickness of 10 random positions of 8 biofilms grown with 4 different bacterial cultures. Statistical significance is indicated with asterisks, * P ≤ 0.05, ** P ≤ 0.01., *** P ≤ 0.001.
